# Biophysical adaptations of circulating tumor cells undergoing metastasis

**DOI:** 10.52601/bpr.2025.250018

**Published:** 2025-12-31

**Authors:** Lanfeng Liang, Xiao Song, Shida Wang, Chwee Teck Lim

**Affiliations:** 1 Mechanobiology Institute, National University of Singapore, Singapore 119077, Singapore; 2 Department of Biomedical Engineering, College of Design and Engineering, National University of Singapore, Singapore 119077, Singapore; 3 Institute for Health Innovation and Technology, National University of Singapore, Singapore 119077, Singapore

**Keywords:** Circulating tumor cells, Mechanobiology, Physical stresses, Mechanical adaptation

## Abstract

Circulating tumor cells (CTCs) are cancerous cells that break away from the primary tumor, enter the bloodstream, and travel to another part of the body. Research into CTCs, particularly their biological phenotypes and molecular mechanisms, has provided critical insights into metastasis and potential therapeutic targets. From a biophysical or mechanobiological perspective, CTCs must undergo biomechanical adaptations to navigate the processes of intravasation, circulation, arrest, and extravasation. These adaptations enable them to interact with blood components and survive in the circulatory system for hours or even days, ultimately facilitating metastatic progression. As research on metastasis within the bloodstream advances, this review explores the mechanobiology of CTCs, emphasizing the cellular and molecular mechanisms that regulate their suspension and adhesion states. Understanding these dynamic behaviors will offer deeper insights into CTC biology and the metastatic cascade.

## INTRODUCTION

Despite significant advances in cancer research and treatment, metastasis remains the primary cause of cancer-related death worldwide (Hanahan 2022). The metastatic process is highly inefficient, and not all patients with a tumor will eventually develop metastatic disease. Estimates from animal studies suggest that a primary tumor can release millions of tumor cells into circulation daily, yet only a minuscule fraction successfully extravasates to seed metastases, with the majority succumbing to the hostile conditions of the circulatory system and ending their cell fate (Butler and Gullino 1975; Gupta and Massagué 2006; Hart and Fidler 1980). Previous studies have demonstrated that not all human cancer cell lines possess the capacity to form metastases when introduced intravenously into immunodeficient NSG mice (Jin *et al*. 2020). While the role of immune function in suppressing tumor progression and metastasis is well established, a critical question remains: beyond immune factors, what additional influences in the circulatory system determine the outcomes of metastasis?

Once cancer cells detach from the primary tumour and enter into the bloodstream, they travel either as single cells or as cell clusters — referred to as circulating tumour cells (CTCs) — until they arrest in the capillary beds. In addition to biochemical factors that influence CTC functions and metastatic potential, these cells are capable of sensing and responding to biophysical stresses through mechanotransduction (Chaffer and Weinberg 2011). In practice, CTCs are subjected to the mechanical stresses within circulation that may persist for hours or even days, influencing their cellular transcription, function and phenotype. For example, mechanical deformation exerted by passage through narrow capillaries can rupture the CTCs’ nuclei, increasing genome instability (Au *et al*. 2016; Jiang *et al*. 2023). Paradoxically, surviving tumor cells may emerge with enhanced cell proliferation, DNA damage repair, and chemoresistance, potentially leading to the development into highly malignant phenotypes (Jiang *et al*. 2023). Advancing our understanding of CTC mechanobiology can provide valuable insights into the metastatic process and uncover potential vulnerabilities that could be targeted to metastasis during circulation.

**Figure 1:**
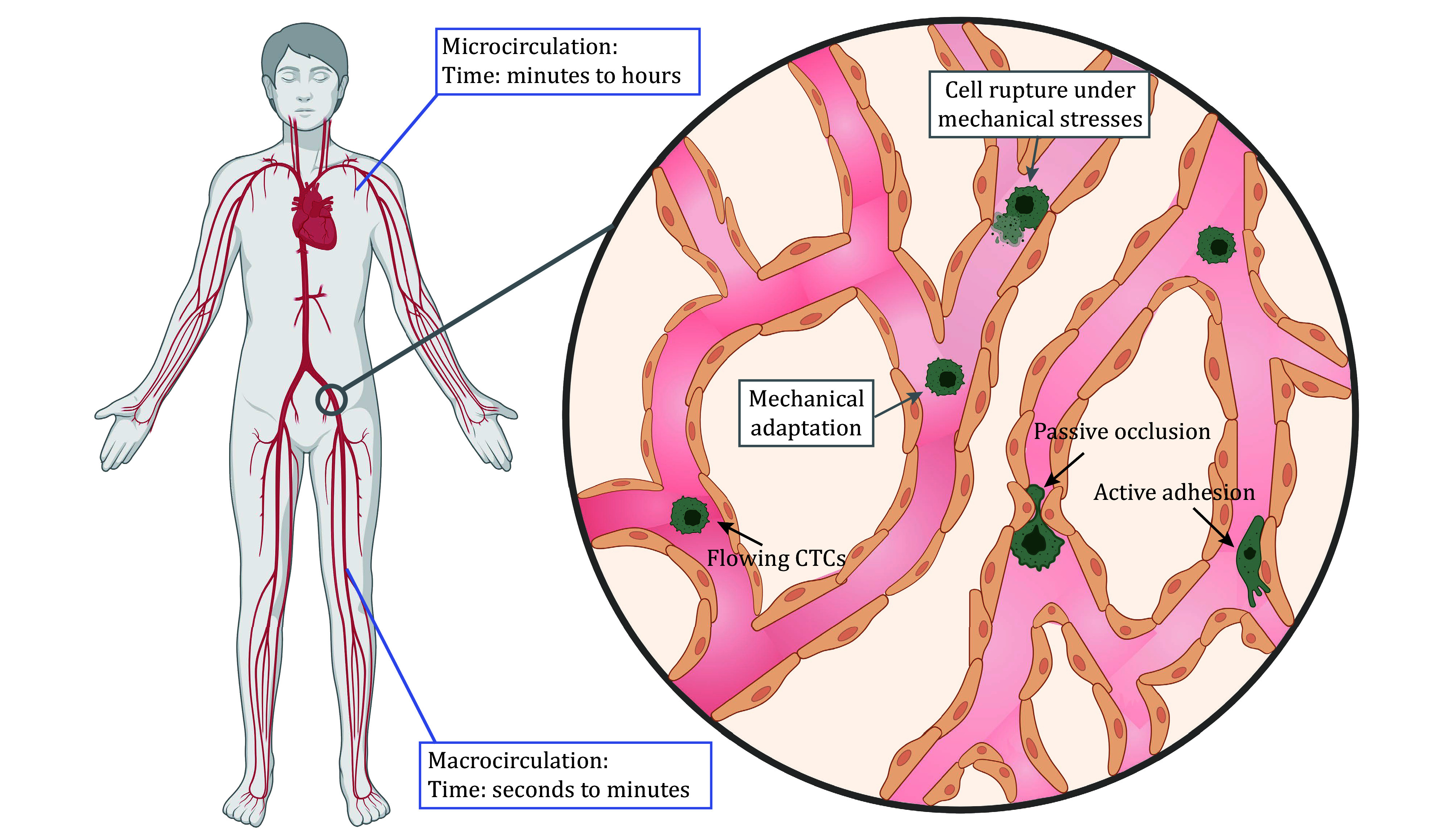
CTCs dynamically traverse the bloodstream, exhibiting either as a suspended or adherent phenotype. Partially created with BioRender.com

This review provides a current overview of advancements in our understanding of cancer metastasis within circulation, with a particular focus on the mechanobiology of CTCs. We emphasize the importance of studying two critical phenotypes of CTCs — the suspended and adhesive states — and their respective mechanotransduction mechanisms and mechanobiological responses (Fig. 1). Finally, we propose directions for future studies to address these underrepresented aspects of metastasis in the circulatory context.

## CHALLENGES AND ADAPTATIONS OF SUSPENDED CTCS IN MACROCIRCULATION

**Figure 2:**
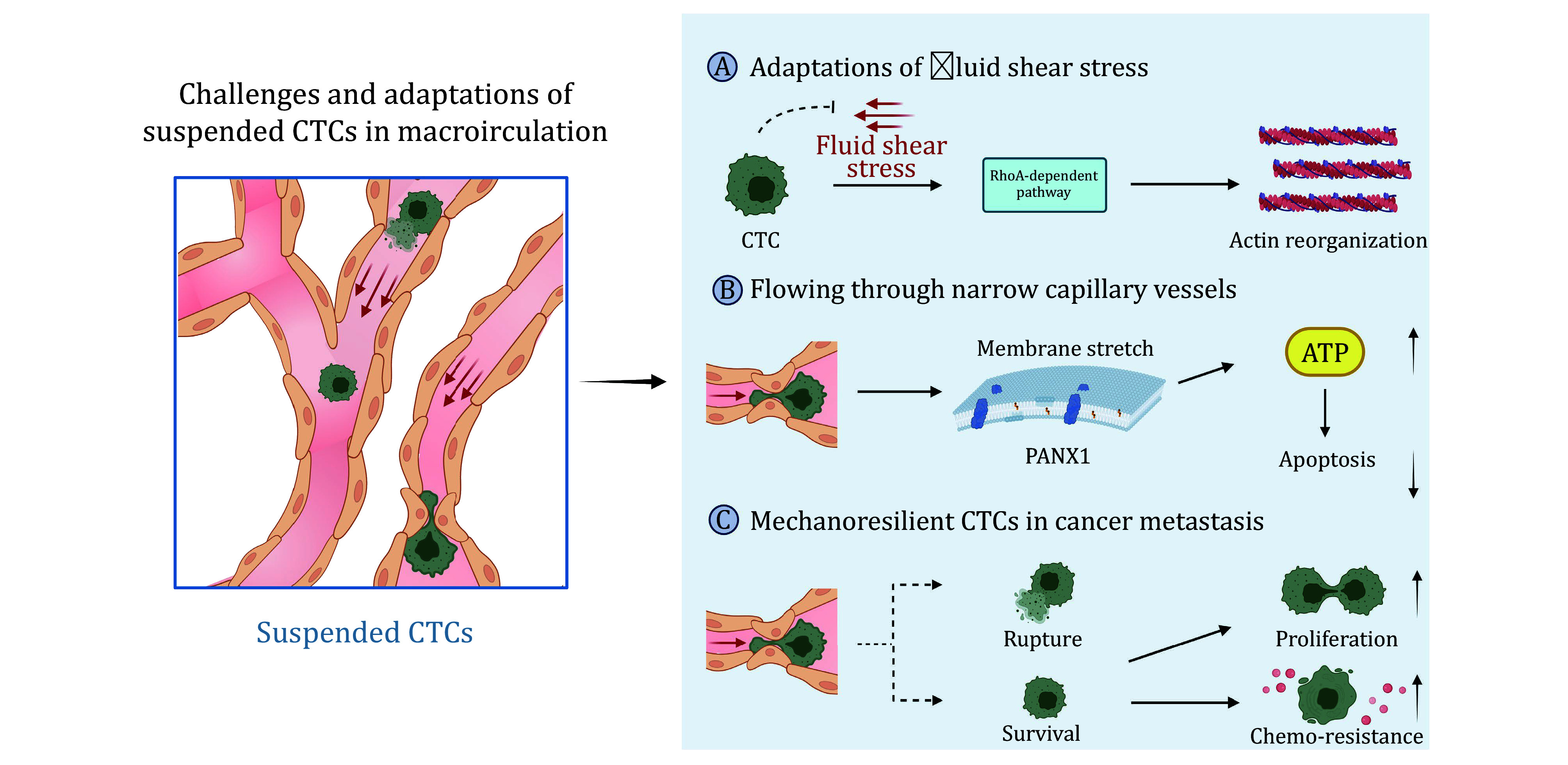
CTCs exist in a suspended state as they traverse the bloodstream, where they are continuously exposed to dynamic biomechanical stresses. To withstand this hostile environment, CTCs must undergo adaptive mechanobiological responses that enable their survival and support subsequent metastatic progression. At the same time, these biomechanical forces act as a selective filter, eliminating vulnerable cells while enriching subpopulations with greater mechanical resilience and metastatic potential. The figure was created with BioRender.com

Cancer cells break away from primary tumors as single cells or cell clusters and intravasate into the lymphatic and/or blood vessels, which provide them with a vascular highway for metastatic dissemination. Although lymphatic spread worsens prognosis, it is considered local dissemination rather than true metastasis (European Working Group for Breast Screening Pathology 2003). Cancer cells within lymph nodes may ultimately enter the systemic venous system or brachiocephalic veins, gaining access to the bloodstream. Hematogenous spread, the primary route of metastasis, enables cancer cells to travel vast distances throughout the body. However, CTCs are exposed to biomechanical forces in blood circulation (Fig. 2), where mechanical cues such as fluid shear stress (FSS) and geometric deformation by blood vessels play a crucial role in influencing the outcome of metastasis (Follain *et al*. 2018; Jiang *et al*. 2021; Varotsos Vrynas *et al*. 2021).

CTCs encounter significant FSS in the vasculature, a key rate-limiting factor for tumor cell survival (Wirtz *et al*. 2011). Shear stress is heterogeneous in the tumor metastasis–related fluid microenvironment. In humans, the mean FSS is approximately 1–4 dyne/cm^2^ in central veins, 10–20 dyne/cm^2^ in capillaries, and 4–30 dyne/cm^2^ in arteries (Huang *et al*. 2018). CTCs have a short lifespan in the bloodstream, with an estimated half-life of 1–3 h, and the majority undergo apoptosis or extravasate within 12 h following intravasation (Meng *et al*. 2004). Exposure to shear stress of 60 dynes/cm^2^ for 4 h resulted in apoptosis, leading to the death of 90% of CTCs derived from various cancers, including breast cancer (MDA-MB-231, UACC-893), lung cancer (A549), and ovarian cancer (2008) during the 24 h post-circulatory incubation (Regmi *et al*. 2017). However, these experiments also revealed that certain cellular features can facilitate the survival of CTCs. For example, knockdown of lamin A/C significantly reduces the resistance of tumor cells to FSS (Mitchell *et al*. 2015). As a mechanism to adapt FSS, cancer cells can activate a RhoA-dependent mechano-adaptive pathway that protects them from plasma membrane damage (Moose *et al*. 2020). In parallel, activation of the c-Jun N-terminal kinase (JNK) signaling pathway has been shown to reduce F-actin organization and cellular stiffness, collectively influencing the survival of suspended tumor cells under shear conditions (Xin *et al*. 2020). HCT116 cells exhibited higher viability and β-catenin expression under high wall shear stress, contrasting with previous studies showing decreased viability and β-catenin inhibition in other colon cell lines (*e*.*g*., SW480, HT29, SW620) under similar conditions (Shaw *et al*. 1997). Interestingly, although we emphasize the survival stress on CTCs caused by FSS, shear stress also upregulates genes associated with brain metastasis and stemness, thereby facilitating subsequent migration and extravasation (Jin *et al*. 2018; Ma *et al*. 2017). Additionally, researchers also found that FSS in the vasculature can select for a rare and highly malignant tumor cell subpopulation (Xin *et al*. 2024). Exposure of suspended tumor cells to an FSS of 10 dyne/cm^2^ for 12 h enhances the ability of cell invasion and the expression of stemness-associated markers in the surviving population. And this subpopulation exhibits enhanced tumorigenic capacity, efficiently establishing both local and metastatic tumors at primary and distant sites compared with the original population. Notably, FSS selectively enriches CXCR4^+^ cells, which display greater resistance to shear-induced apoptosis. As a mechanosensitive adaptation, tumor cells modulate their phosphorylation state, but not the total expression of PI3K to withstand FSS.

In addition to the shear stress encountered during circulation, CTCs are subjected to a variety of mechanical constraints within the microvascular network, particularly when CTCs transit through narrow blood capillary (Paizal *et al*. 2021). The rapid flow of CTCs through narrow capillaries within seconds or minutes imposes substantial mechanical stress, as many capillaries (typical diameters of 8 μm in human) are smaller than the cell nucleus (Müller *et al*. 2008), forcing pronounced stretching and deformation of both the plasma membrane and the nucleus. This large stretching also causes the activation of part membrane-local channel, affecting the survival of CTCs. For example, the cell-surface channel protein pannexin-1 (PANX1) is involved in apoptosis and cancer progression (Furlow *et al*. 2015). The activation of PANX1 channels in response to capillary constrictions led to the release of extracellular ATP and subsequent activation of cell-surface purinergic receptors such as P2Y, which were involved in survival signaling during deformation-induced injury. Consequently, P2Y receptor activation inhibited apoptosis induced by mechanical stress, thereby contributing to metastatic efficiency. Metastatic breast cancer cells with a PANX1 channel-activating mutation gained a survival advantage due to an increase in the release of ATP, which was modulated via PANX1 when the breast cancer cells became lodged in the microvasculature. Notably, CTC clusters, the multicellular aggregates of circulating tumor cells, have been shown to possess higher metastatic potential, with over 90% of clusters containing up to 20 cells successfully navigating 5- to 10-μm constrictions, even within whole blood (Ring *et al*. 2023; Sayed *et al*. 2024). Microfluidic devices designed to mimic human capillary constrictions have revealed that these clusters can rapidly and reversibly reorganize into single-file, chain-like configurations, significantly reducing their hydrodynamic resistance (Au *et al*. 2016). In xenotransplantation studies, human CTC clusters were observed to undergo similar reorganization and successfully traverse capillary-sized vessels *in vivo* in zebrafish models (Chen *et al*. 2022). These findings suggest that CTC clusters may play a more substantial role in tumor dissemination than previously recognized. As a potential therapeutic target for the prevention of tumor metastasis, some preliminary experiments also demonstrated that these clusters could be disrupted during transit by drugs that alter cellular interaction energies (Liu *et al*. 2024).

The mechanical forces acting on CTCs also have profound effects on the nuclear architecture, resulting in the production of nuclear blebs after the rupture of the nuclear lamina (Irianto *et al*. 2017). These blebs facilitate the exchange of nucleocytoplasmic contents and mis-localize nuclear repair factors, impairing the efficiency of nuclear envelope (NE) repair. As a consequence, the repair of the NE may be delayed, leading to the accumulation of DNA damage. This delay in DNA repair is a key factor contributing to increased chromosomal instability, which promotes tumor heterogeneity and enhances the ability of cancer cells to adapt to new microenvironments. Although some studies do not specifically focus on CTCs, millisecond-scale deformation of the cell nucleus can induce wrinkling and transient disassembly of the nuclear lamina, leading to local detachment of lamina-associated chromatin domains and a reduction in both histone H3 lysine nine trimethylation and DNA methylation (Song *et al*. 2022). Meanwhile, our lab observed that mechanical deformation eliminates a large proportion of CTCs, but it also enriches a resilient subpopulation with marked resistance to squeezing-induced cell death (Jiang *et al*. 2023). This resilient subpopulation is characterized by the upregulation of pathways involved in cell proliferation and DNA damage response, promoting the survival and proliferation of CTCs under mechanical stress with enhanced resistance to chemotherapy, thereby complicating treatment strategies. Overall, these adaptations may be attributed to differences in shear and confinement conditions experienced by CTCs, suggesting distinct regulatory mechanisms in intracellular signaling. The understand of the adaptations of the shear and deformation will increase the strategy that prevent the early development of tumor metastasis.

## MECHANOBIOLOGICAL INSIGHT OF ADHESIVE CTCS IN MICROCIRCULATION

**Figure 3:**
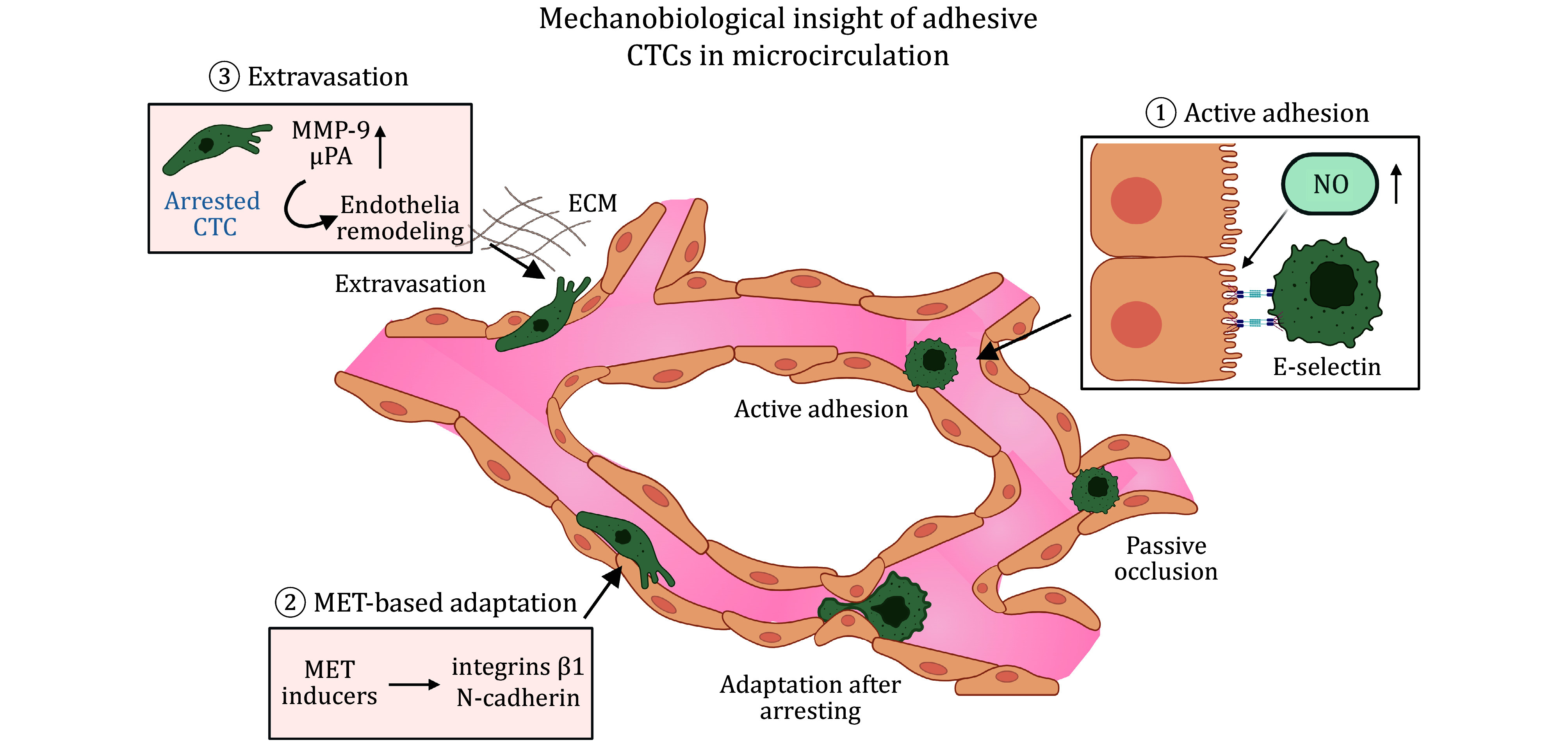
CTCs endure extreme biomechanical stresses as they transition from a suspended to an adherent state during metastasis. Their arrest within microvessels occurs through passive occlusion or active adhesion, processes governed by vessel geometry, shear flow, and adhesion molecules such as E-selectins and integrins. MET-associated adaptations further enhance their adhesive capability. To establish successful metastases, adherent CTCs must breach endothelial junctions and undergo extravasation, a process facilitated by proteolytic enzymes such as MMP and μPA. The figure was created with BioRender.com

The ability of CTCs to survive in circulation and establish secondary tumors hinges on their capacity to adhere to the endothelium, extravasate, and colonize at distant tissues (Gensbittel *et al*. 2021). This transition from a suspended to an adhesive state represents a critical juncture in the metastatic cascade, influencing whether CTCs are eliminated by hemodynamic forces or succeed in forming metastatic lesions. Multiple biomechanical and molecular mechanisms regulate this process, including cell adhesion dynamics, mechanical stress adaptation, and endothelial remodeling (Fig. 3) (Lin *et al*. 2021).

CTC arrest occurs through two primary mechanisms: passive occlusion and active adhesion (Gensbittel *et al*. 2021). Passive occlusion results from the mechanical lodging of CTCs in capillary beds due to topological disorder and size constraints, particularly in the lungs, liver, and brain, where dense microvascular networks contain capillaries with irregular diameters and tortuous geometries that increase the likelihood of entrapment (Entenberg *et al*. 2018; Headley *et al*. 2016; Kienast *et al*. 2010). This phenomenon is influenced by vessel geometry and flow dynamics, with bifurcations and regions of low shear stress serving as preferential sites for CTC retention (Casas-Arozamena *et al*. 2021; Cui *et al*. 2021). These microvascular constraints impose profound mechanical stress on CTCs. As tumor cells are forced through narrow constrictions, they undergo severe cytoplasmic and nuclear deformation, which can result in nuclear envelope rupture, chromatin reorganization, DNA damage, and even apoptosis (Headley *et al*. 2016; Paizal *et al*. 2021). Nevertheless, metastatic CTCs exhibit remarkable resilience. They dynamically adapt by reorganizing the cytoskeleton, modulating nuclear lamina composition, and activating DNA repair pathways to counteract deformation-induced damage (Gensbittel *et al*. 2021; Paizal *et al*. 2021).

In parallel, active adhesion mechanisms provide CTCs with the molecular means to stabilize their arrest under flow conditions and resist detachment. Tumor cells preferentially adhere to regions of intact microvessels with elevated endothelial nitric oxide (NO) production, such as curved portions (Zhang *et al*. 2016). And highly metastatic tumor cells generally display greater adhesive capacity than their low-metastatic cells. This active adhesion process begins with weak, transient bonds mediated by selectins and CD44, which facilitate initial tethering and rolling interactions along the endothelial wall (Follain *et al*. 2018; Osmani *et al*. 2019). These low-affinity contacts serve as a prelude to the formation of stronger, integrin-dependent adhesions. Integrins such as αvβ3, β1, and α5β1 engage endothelial ligands, including fibronectin deposited under shear stress, enabling CTCs to form firm attachments (Chen *et al*. 2016). Additionally, selectins, particularly E-selectin and P-selectin, contribute to the initial transient attachment, enabling rolling along the endothelial surface before integrin-mediated stabilization occurs (Aigner *et al*. 1998; Laferrière *et al*. 2001; Li *et al*. 2019). P-selection increases the preferential interaction between tumor cells and peritoneal mesothelium under ascitic FSS (Li *et al*. 2019). Intravital imaging studies reveal that adhesion must overcome a critical mechanical threshold — approximately 80 pN of binding force — to resist vascular shear forces and stabilize intravascular arrest (Follain *et al*. 2018). Shear flow itself is not merely a hostile force but also a regulator, as permissive shear profiles facilitate adhesion while excessive shear detaches weakly bound cells. Moreover, the adhesive repertoire of CTCs is often reinforced by platelets, which cloak tumor cells, protect them from immune surveillance, and provide additional integrin and selectin mediated contacts with the endothelium (Gensbittel *et al*. 2021; Labelle *et al*. 2014). The interplay between passive and active mechanisms is crucial, as it determines whether CTCs remain transiently trapped or develop stable interactions that support extravasation.

Once arrested, CTCs must withstand biomechanical forces such as FSS stress and compression from the endothelial wall (Mohammadi and Sahai 2018). Metastatic cells demonstrate a higher resilience to these stresses compared to non-malignant cells, largely due to their ability to remodel the cytoskeleton, regulate actomyosin contractility, and activate mechanosensitive pathways such as YAP/TAZ and RhoA-ROCK (Lee *et al*. 2017; Paizal *et al*. 2021). For example, in lymphatic flow, low wall shear stress specifically activates YAP1 via the ROCK–LIMK–cofilin pathway, leading to TEAD-dependent gene expression that promotes migration and metastasis (Lee *et al*. 2017; Zhao *et al*. 2010). Tumor cells with constitutively active YAP overexpression exhibited the ability to migrate through the capillary plexus, re-enter systemic circulation, and establish secondary lesions in the brain (Benjamin *et al*. 2020). And as a dissemination mechanism of medulloblastoma, tumor cells utilize mechanosensitive ion channel PIEZO2 to sense FSS and subsequently promote actomyosin contractility–dependent translocation of GLUT1 to the plasma membrane (Min *et al*. 2025). Additionally, mechanosensitive ion channels such as PANX1 and Piezo1/2 enable CTCs to translate membrane stretch and shear forces into survival signals, sometimes conferring resistance to apoptosis (Coste *et al*. 2010; Laird and Penuela 2021). Nuclear adaptations also contribute to the activation of genes associated with epithelial-to-mesenchymal transition (EMT) (Chen *et al*. 2018). EMT enhances the survival and adhesion of arrested CTCs within the vasculature, enabling them to resist shear stress and establish firm interactions with the endothelium (Brabletz *et al*. 2018). EMT-driven cytoskeletal remodeling reduces intracellular stiffness, allowing CTCs to endure mechanical deformation and hydrodynamic forces in circulation. Additionally, EMT upregulates mesenchymal adhesion molecules such as integrins β1 and N-cadherin, which strengthen CTC attachment to the endothelium, preventing their detachment under blood flow (Brabletz *et al*. 2018; Chen *et al*. 2018).

Extravasation is the next critical phase following CTC arrest, where CTCs must traverse the vascular barrier and invade the surrounding stroma (Chaffer and Weinberg 2011). While the extravasation process shares mechanistic parallels with immune cell transmigration, CTCs must overcome unique biomechanical challenges. Unlike immune cells, which undergo transendothelial migration via paracellular or transcellular pathways (Wittchen 2009), CTCs frequently induce endothelial remodeling to facilitate their exit. This is mediated by the upregulation of proteases such as MMP-9 and urokinase plasminogen activator (uPA) (Chiang *et al*. 2016), which degrade the basement membrane and extracellular matrix, clearing a path for invasion (Egeblad and Werb 2002). Additionally, CTCs employ actin-rich protrusions such as invadopodia, which exert localized forces on the endothelium and basement membrane. These protrusions not only facilitate ECM degradation but also generate the traction forces required to breach endothelial junctions (Micalizzi *et al*. 2017). Live imaging studies reveal that endothelial cells can also undergo cytoskeletal reorganization in response to CTC contact, leading to junctional loosening and the formation of transendothelial pores that expedite transmigration (Follain *et al*. 2018).

Research also suggests that CTC clusters have a higher extravasation efficiency than single CTCs, likely due to cooperative signaling and increased resistance to mechanical stress (Haeger *et al*. 2014). Within these clusters, intercellular junctions provide mechanical stability, while differential expression of adhesion molecules allows division of labor between cells at the invasive front and those maintaining cluster integrity (Mazzone and Bergers 2019). For heterogeneous CTC clusters, platelets play a crucial role in EMT-related shielding, arresting CTCs from immune surveillance and enhancing their adhesive properties (Chiang *et al*. 2016; Micalizzi *et al*. 2017). Platelet-derived transforming growth factor-beta (TGF-β) has been shown to reinforce mesenchymal traits in CTCs, boosting their invasive capabilities. Additionally, platelet-CTC aggregates facilitate firm adhesion through integrin-mediated interactions, increasing the likelihood of successful extravasation (Weigelt *et al*. 2005). These mechanisms allow the CTC cluster to successfully navigate the extravasation process, overcoming mechanical resistance and immune clearance to establish metastatic colonies in distant tissues. Following vascular extravasation, CTCs face the challenge of adapting to new tissue environments. Here, phenotypic plasticity through EMT and mesenchymal-to-epithelial transition (MET) becomes critical, as mesenchymal traits facilitate migration and invasion, while epithelial characteristics support proliferation and colony formation (Chen *et al*. 2018; European Working Group for Breast Screening Pathology 2003). The interactions between CTCs and resident stromal cells, immune components, and extracellular matrix elements further dictate metastatic potential. Notably, CTC clusters exhibit enhanced adaptability, leveraging intercellular cooperation to withstand stress and optimize survival in secondary sites (Mazzone and Bergers 2019). As research continues to unravel the complexities of extravasation and subsequent metastatic colony, new therapeutic avenues targeting adhesion molecules, mechanosensitive pathways, and endothelial interactions emerge as promising strategies to hinder metastatic progression.

## SUMMARY AND PERSPECTIVE

Over the past two decades, researchers have applied diverse engineering approaches to successfully isolate rare CTCs (typically fewer than 20 cells per milliliter of blood) from millions of hematologic cells, thereby advancing their clinical application (Hou *et al*. 2013; Lin *et al*. 2021). These technological developments have greatly enabled comprehensive characterization of CTCs at the genomic, transcriptomic, epigenomic, and proteomic levels, and have even established CTC analysis as a promising liquid biopsy approach in clinical diagnostics (Gu *et al*. 2024; Yang *et al*. 2025). Nevertheless, the investigation of CTCs should not stop at this stage. During metastasis in the circulation, CTCs experience biomechanical microenvironments that are highly distinct from those of primary tumors. Such mechanical forces profoundly affect CTC survival and may either hinder or terminate the metastatic cascade. Inversely, these stresses might provide a selection for those metastasizing cancer cells, enriching their subpopulation with highly metastatic potential, which challenges the therapy of metastatic disease (Liang *et al*. 2024). Understanding how CTCs sense and adapt to these biomechanical cues, and what functional advantages they acquire after surviving such stresses, is essential for finding the potential clinical targets.

In this review, we highlight the two phenotypes of CTCs, suspended and adhesive (Fig. 1). The most revealing findings of the past decade on CTCs have primarily focused on suspended CTCs (Cognart *et al*. 2020; Jiang *et al*. 2023; Regmi *et al*. 2017). However, the residence time in circulation only reaches up to seconds or minutes. Arresting CTCs may spend hours or even days before escaping the circulation system (Varotsos Vrynas *et al*. 2021), greatly increasing the likelihood of converting and retaining these mechanical signals into mechanical activation. This mechanotransduction specificity might provide a new potential strategy targeting CTCs and stop metastasis in an early stage. For example, advances in intravital microscopy have revealed that certain cancer cells migrate along the walls of narrow blood vessels instead of passively flowing with the bloodstream (Headley *et al*. 2016; Wu *et al*. 2021; Zhao *et al*. 2023). This migratory behavior is likely associated with subsequent extravasation events. Unlike conventional two-dimensional migration observed on culture substrates, this form of motility requires CTCs to adapt to a foreign and dynamically changing mechanical environment, which can induce sustained activation of specific mechanotransduction pathways, such as PIEZO1 (Min *et al*. 2025; Silvani *et al*. 2025) and YAP (Lee *et al*. 2017; Shah *et al*. 2025). YAP regulates cell migration and invasion by modulating Rho-GTPase activity (Shah *et al*. 2025). However, it remains unclear whether inhibition of the YAP or PIEZO1 pathway can effectively suppress this motility or even prevent metastatic dissemination. Furthermore, it remains unclear whether these mechanical stimuli modulate tumor cell sensitivity or resistance to specific chemotherapeutic agents. Although several clinical trials target CTCs, for example, using the PLK1 inhibitor BI 2536 to prevent CTC intravasation (Donato *et al*. 2020) and interfering with VEGFR signaling (Groppa *et al*. 2018), our understanding of how mechanical cues contribute to CTC metastasis remains limited. Alternatively, as argued here, investigating both phenotypes and their underlying mechanobiological mechanisms will likely become a crucial component of future cancer management. Encouragingly, as our understanding of metastasis advances, confidence in overcoming metastatic disease continues to grow.

## Conflict of interest

Lanfeng Liang, Xiao Song, Shida Wang and Chwee Teck Lim declare that they have no conflict of interest.
